# Shared leadership team: the creation, journey, lessons learned

**DOI:** 10.3389/fpubh.2026.1695469

**Published:** 2026-03-17

**Authors:** Tasha Joshua, Reina Lopez, Jordan S. Wilson, Marie Owino, Endaisia Love, Leah Schinasi, Melissa J. Kaufman, Elizabeth Waetzig, Gina S. Lovasi, Renee’ H. Moore

**Affiliations:** 1 Dornsife School of Public Health, Drexel University, Philadelphia, PA, United States; 2 Change Matrix, Las Vegas, NV, United States

**Keywords:** culture, equity, higher education, leadership, shared leadership

## Abstract

Drexel University’s Dornsife School of Public Health (DSPH) upholds its founding belief that health is a human right. In pursuit of this commitment, DSPH has made addressing racism and discrimination a core public health priority through intentional diversity, equity, inclusion, and belonging (DEIB) efforts for all. To challenge traditional hierarchical structures that have excluded the voices and decisions of many, the school established a Shared Leadership Team (SLT) in 2023, comprised of students, faculty, and staff across units to guide and implement DEIB initiatives collaboratively. This article documents the SLT’s development and the founding of the Office of DEIB, highlighting successes, challenges, and strategies to maintain momentum amid institutional change and national political changes. Featured initiatives include a school-wide climate survey, shared DEIB definitions, and the growth of the IDEA Fellows program. We reflect lessons learned through a series of vignettes, demonstrating engagement during low-morale periods, and the value of shared power in creating a more inclusive, equitable culture within academic public health.

## Drexel University’s Dornsife School of Public Health

Drexel University’s Dornsife School of Public Health (DSPH) was founded on the belief that health is a human right, and to achieve this, social justice must be pursued. DSPH has a commitment to address racism and discrimination as a public health threat throughout research, practice, and training, with the intent to eliminate unequitable practices and policies within the school. Traditional centralized authority, hierarchical structure, and top-down approaches to decision making present an ongoing challenge to create and sustain an inclusive environment where all feel they belong and are valued.

To address this challenge, DSPH developed a shared leadership team consisting of students, faculty, and staff across 14 units or departments to inform and enhance DEIB programming within the school. The Shared Leadership Team (SLT) facilitates diversity of thought, perspective, and interest by intentionally bringing everyone to the table, ensuring each individual is seen and included, and that their perspective, recommendations, and input are valued and respected.

In this article, we chronicle the journey of developing the SLT and the office of Culture, Community, and Opportunity (CCO) at DSPH through a series of vignettes, highlighting the journey through the voices of students, faculty, staff, and other partners that have worked toward these goals and persevered through the many challenges of creating a more equitable climate and future within our community. These insights provide context for the development of the shared leadership model (1), its evolution from the DSPH Action Plan in 2020, and shed light on the challenges of maintaining the SLT during a period of transition (2).

The structure and impact of the SLT will be highlighted, outlining how a multi-level approach was created, including the process of providing clarity around key concept definitions and methods to increase engagement in an ever-changing environment. Lastly, we will provide an overview of how we adhere to the current federal administration’s orders to eliminate such programming and our process of re-evaluating and maintaining the mission, vision, and values of DSPH.

The Leading through Change Framework was used to guide and inspire the shared leadership journey within DSPH. Leading through Change Framework was developed by Change Matrix to guide systemic change by combining elements of the Adaptive Leadership Framework (3), a diagnostic and behavioral approach for tackling adaptive challenges, and The Heart of Change (4), a process-driven model emphasizing emotional engagement and a collective sense of urgency while moving through elements of a change process. The iterative Leading through Change Framework, includes steps that are non-sequential and can be repeated and refined. The steps of the Leading through Change framework are taken in the categories: creating conditions for change, engaging diverse voices for action, and implementing and sustaining (4). Together, these steps support systems to recognize and address adaptive challenges, identify, acknowledge and include cultural norms, mental models, and competing values, while creating a sense of urgency to build a guiding coalition. Inspired by this framework, DSPH aligned with the steps to lead through change and used a shared approach to DEIB efforts.

## Associate Dean established

In 2020, former DSPH Dean, Ana Diez-Roux, MD, PhD, MPH named Scarlett Bellamy, ScD the first Associate Dean of Diversity, Equity, and Inclusion (DEI) and the Action Plan to Enhance Diversity, Inclusion, Equity, and Anti-Racism (also referred to as the Anti-Racism Action Plan, AAP) was created. As part of the AAP, the DSPH Anti-Racism Task Force was developed to create and sustain structure, policies, and culture changes while promoting the academic success of diverse scholars and the production of scientific knowledge that eliminates inequities. This task force was composed of individuals in administrative leadership roles within DSPH such as the dean, associate deans, department chairs, and school wide center directors.

*“The Anti-Racism taskforce operated with a top-down approach and was comprised of school leadership, including the department chairs, associate deans, chair of the Executive Committee of the Faculty (ECF), and chair of the Staff Coordinating Committee (SCC). I was invited to be on the task force based on my role at the time as Chair of the SCC. About a year later when I rolled off as Chair of SCC, I also rolled off the task force. I would have liked to remain on the committee, but under that structure, there were limited seats and since I was no longer chair of SCC, there was no longer a seat at the table. I completely understood this and tried to support the work from the sidelines as I was able*.” Melissa J. Kaufman, DSPH Associate Dean for Education & Former Chair of the Staff Coordinating Committee.

The Action Plan (2020–2023) identified goals of creating an inclusive culture; standards for infusing DEIB research, practice, and academic programming into DSPH; and forming intentional, equitable and inclusive partnerships. The Action Plan identified four pillars to guide the DSPH community: (1) creating a culture and environment of inclusivity and antiracism, (2) maximizing racial diversity of faculty, staff, and students, (3) academic programming, and (4) research practice and partnership. While the work of the Anti-Racism Taskforce initiated important conversations about systemic racism and the importance of an equitable and inclusive culture, the individuals selected to advocate on behalf of the school did not represent its population. The task force consisted of disproportionately white members within executive leadership roles, missing the vital voices of students, faculty and staff across the school. With the Action Plan and Taskforce, DSPH made progress towards the four pillars; however, it was clear that other initiatives were needed to continue movement.

## TAE grant and partnering with RWJF

The Transforming Academia for Equity (TAE) Grant through the Robert Wood Johnson Foundation (RWJF) offers a network of peer institutions and Adaptive Change Specialists (ACS) opportunities to share insights and learn from one another while advancing system-level equity, diversity, and inclusion efforts within academia and allowing the school to build on the work begun under the four pillars of the Action Plan, specifically with health equity research. With this TAE grant, DSPH was provided with funding to dedicate staff and protected time to advance the AAP.

As one of seven institutional recipients of the initial TAE grant, DSPH set aims to (1) Assess barriers and facilitators to advancing health equity research, (2) Develop and implement a change plan to address the barriers and support facilitators identified, and (3) participate in peer-learning communities with other funded sites to improve and support successful implementation. One of our first initiatives under aim 1 was to administer faculty focus groups to evaluate the facilitators and barriers of health equity research at DSPH. This part of the grant focused on health equity research within DSPH, ensuring that there was a collaborative culture where research and researchers were supported, valued, and thrived. During these focus groups, we provided an opportunity for faculty to express themselves freely.

*“In our initial group meetings, we spent considerable time discussing the goals of the project and, importantly, what we meant by health equity. We explored how faculty (especially junior faculty) could be supported in conducting equity-focused research. We also spent a great deal of time discussing the importance of language and communication and deriving very specific and thoughtful definitions and conceptualizations of health equity, thinking not just about what currently exists, but also about what could be, in the absence of barriers. Given the nature of these discussions, which often touched on structural obstacles to conducting health equity work, it was essential that we have a group environment characterized by trust and honesty. Transparency was critical for making meaningful progress and having candid conversations. The fact that I felt comfortable being vulnerable, and talking about sensitive issues with the other faculty in the room was essential.”* Leah Schinasi, DSPH Assistant Professor, TAE Guiding Team Member.

*“I was initially brought into the Office to reanalyze the focus group data. The process of qualitative analysis is iterative and involves multiple coders, which enhances the credibility of the analysis. I (staff) worked alongside a student to analyze the data, and we held several meetings to discuss the focus groups. This was my first experience with shared leadership. The student and I were both able to contribute to the analysis and constructively challenge each other's opinions. This led to a comprehensive analysis and better understanding of the perspective we brought to the work.”* Jordan S. Wilson, DSPH Staff, Office of Culture, and Community and Opportunity.

### Lessons learned

Hosting the aim 1 focus groups provided DSPH with insight into the faculty experience and how to advance health equity work. They provided a space for members to express themselves without consequence and to discuss the barriers and facilitators they faced as faculty members conducting health equity research. Some themes that emerged from the data were the time required for equity research, the social/financial capital needed to make an impact, and the need for safety/security of the people doing this research.

When analyzing the focus group data, we prioritized coders who could provide multiple perspectives. We felt it was important to include perspectives from people with diverse backgrounds and to give a voice to those who may not traditionally have a prominent role in the academy (e.g., students, staff). The diverse perspectives and coders also helped with interpretation and facilitating different people being at the table and in the conversations about how to better facilitate and advance community engagement and health equity work at DSPH.

## Shared Leadership Team (SLT)

During part 2 of the TAE grant, DSPH established an Office of Diversity, Equity, Inclusion and Belonging, now named the Office of Culture, Community, and Opportunity, (CCO), and developed a Shared Leadership Team (SLT) of students, faculty, and staff across DSPH to inform and enhance DEIB initiatives. The SLT includes those (positions) who were initially on the Antiracism Action Task Force which greatly mirrors the Dean’s Executive Committee but also triples the membership to include students, staff, and a broader range of faculty. The SLT is a standing committee within DSPH, that collaboratively informs and provides insight when decision making towards various efforts impacting the school. The Office of CCO facilitates discussions with SLT to gather a multifaceted feedback and ideas for further discussion during Dean’s Executive Committee meetings, promoting the ideas and thoughts of the larger school community. Programming that the SLT deliberates, and influences has included our school-wide climate survey, shared DEIB definitions, and the expansion of the Impact and Development through Engagement and Advocacy (IDEA) student fellowship program and strategic planning goals.

Leveraging varying perspectives, equity initiatives are openly discussed, and challenges within the school are explored. The SLT aims to share the social and moral responsibility of DEIB work and collectively carry out informed decision-making. The SLT meets quarterly and within smaller taskforces throughout the year to provide guidance and insights from the DSPH community regarding DEIB initiatives and culture and community within DSPH. Since Spring 2023, we have grown the SLT with an emphasis on including more student and staff voices to improve our awareness and programming, encouraging members from each department to join. With a larger leadership team of over 50 individuals, the composition of the SLT is constantly changing as team members move in and out or expanding as we realize units or perspectives have not been represented; as examples, a full-time online student and a staff academic advisor were recently added to SLT. As a result, we are constantly building community and exploring new perspectives to enhance our awareness of members’ experiences.

*“Working with the team has given me an opportunity to interact with DSPH faculty, staff, and other students while also focusing on my research. During this time, I have gained more appreciation for faculty and staff and a better understanding of their stance and views on creating a more equitable environment for all through working with the Office of DEIB and the Shared Leadership Team. The Shared Leadership Team offers a variety of different voices, from student fellows, faculty, staff, and individuals from the TAE grant. The meetings offer a safe place to share how individuals are navigating the current climate within higher education and public health while also giving attendees an opportunity to provide input into strategies and initiatives around creating a more equitable environment within DSPH. All attendees, regardless of position they hold within DSPH, are welcome to share.”* Tasha Joshua, DSPH DrPH Student.

*“As a student, I was initially hesitant to speak up in rooms full of titles and tenure, but I quickly realized my perspective was not just valuable, it was necessary. Working alongside people with different roles and backgrounds—not out of obligation, but because we all genuinely believed in the mission—showed me that meaningful change isn’t dictated from the top but forged through collective commitment.”* Marie Owino, DSPH MPH Student and IDEA Fellow.

*“I joined the Office during a transitional period when it was moving from a hierarchical model to a shared leadership model. Although I was not part of the original structure, I am familiar with the strengths and weaknesses of both models. One strength of the hierarchical model is that decision-making is more streamlined and faster. However, a potential weakness is the disconnect between leadership and those doing the work or those affected by the decisions. The transition to a shared leadership model was a meaningful step forward. It offered an invaluable opportunity for students, staff, and faculty in various roles to collaborate and actively participate in shaping the climate of DSPH.”* Jordan S. Wilson, DSPH Staff in the Biostatistics Scientific Collaboration Center and the Office of Culture, Community & Opportunity.

*“In fall 2024, our work evolved into a shared leadership team. While the core themes of our discussions remained the same, the larger group brought a broader range of perspectives that expanded the scope of our conversations. Around this time, our Dean stepped down and was replaced by an interim Dean, prompting further reflection on the meaning and importance of institutional commitments to equity-centered work. As a group, we circled back to questions around language and implementation. The different perspectives in the room often made me quieter and reflective. However, I believe that these conversations with the larger group, including school leadership, were important for engaging conversations about the structural factors that are critical for fostering an academic environment in which health equity research is not only possible, but also supported and valued.* Leah Schinasi, DSPH Assistant Professor, TAE Guiding Team Member.

*“At Dornsife School of Public Health (DSPH), the ACS [Adaptive Change Specialist] team supported the shared leadership team by starting with the individuals who would convene that team. They explored their own shared vision of equity for the initiative and for the school. The effort to build a shared leadership team started with the recognition that early career faculty did not have a significant or representative voice on the many efforts to address diversity, equity, inclusion, and belonging at DSPH. Engaging their learning in focus groups and site visits provided contextual information and input on the direction of the TAE effort and the new office of DEIB. What started with engaging early career faculty and students expanded to an invitation to other DEIB efforts to join the leadership team creating a more school wide approach.”* Elizabeth Waetzig, TAE Adaptive Change Specialist.

### Lessons learned

By engaging with the SLT in quarterly meetings, the office gained a better understanding of DSPH members’ experiences and created a space for hard conversations. The Office of CCO quickly learned that the SLT meetings offered community in uncertain times and a way for members to process challenges together that often worked on in silos. As the SLT grew, managing team projects became laborious considering the various perspectives and expectations of the members. Exploring ways to prioritize the work while remaining realistic about the capacity of the team was emphasized. We met this challenge through support from our ACS and external partners to best facilitate difficult topics and raw emotions. Creating a space for more open, transparent, and intentional conversation became a focus of the meetings, and this was accomplished through exercises focused on expressing points of view and sharing concerns in a challenging climate. The varied points of view helped to ensure the priorities set were beneficial to the entire school. Future work entails outlining SLT responsibilities and working groups to support progress with the CCO goals in the new DSPH Strategic Plan. This will ensure that more voices are being heard, and the work is shared in a more equitable and efficient manner.

## Defining our work

In 2023, the position of Associate Dean of DEI became the Associate Dean of DEIB, recognizing the importance of belonging within DSPH and the Office of Diversity, Equity, Inclusion, and Belonging was established, both changes under the new leadership of Reneé Moore, PhD. One of the first large scale projects of the SLT was to craft unified DSPH definitions of the words: diversity, equity, inclusion, and belonging. In completing this task, the SLT collaborated and drafted statements representative of their values and detailed how these words should be embraced and implemented within the school. The goal for these definitions is to serve as an anchor for the work and programming produced by DSPH [see Supplementary Data Sheet S1 for definitions.]

*“Upon establishing the Office of DEIB, I immediately wanted the SLT to establish an updated vision and mission for DEIB at DSPH. However, our ACS asked had we together as DSPH defined the four words: diversity, equity, inclusion, and belonging. We had not since I joined DSPH in 2021, and so this became our first large scale project as a SLT. It had many challenges and took almost six months longer than I expected. However, we did it! And we did it incorporating voices of students, staff, and faculty. We even received input during the new student orientation.”* Reneé H. Moore, DSPH Associate Dean of the Office of Culture, Community, and Opportunity.

*“Defining diversity, equity, inclusion, and belonging was the SLT's first actual test of collaboration. Even though we use these words daily, we do not all use them or think about them the same way. This meant the SLT had to figure out how to work together to reach consensus, which wasn't always easy, and it took time for everyone to feel ok with the definitions.”* Jordan S. Wilson, DSPH Staff, Office of Culture, Community and Opportunity.

*“The effort to refine the school’s definitions of DEIB was one of the most impactful projects I contributed to as part of the shared leadership team. Language matters deeply in this work; without shared understanding, progress stalls. We facilitated workshops and discussions to help the community engage with terms like “equity,” “belonging,” and “antiracism” in ways that resonated across departments and identities. These conversations weren’t always easy, seeing as people came with vastly different perspectives, but by creating space for honest dialogue, we helped build a common vocabulary that allowed for more productive and courageous conversations about where the school was falling short and how to move forward. Crafting the definitions required us to wrestle with tough questions: What does true inclusion look like here? How do we hold ourselves accountable? The process was iterative, but it reinforced the importance of grounding DEIB work in both clarity and purpose.”* Marie Owino, MPH Student and IDEA Fellow.

*“When you bring diverse groups into shared leadership, language is often an important initial consideration. A first and important milestone of the shared leadership team was to agree on definitions of diversity, inclusion, equity, and belonging. And as with so many complex challenges, the leadership team has had to adapt their efforts to a rapidly changing environment in 2025.”* Elizabeth Waetzig, TAE Adaptive Change Specialist.

### Lessons learned

Crafting the definitions took 8 months of hard work and was facilitated through integration of technology-enhanced audience interaction (5). The biggest challenge was coming to a united decision of what these terms meant and what should be included in these statements. In the process of determining the definitions, members of the SLT engaged in critical conversations and learned about what it means and looks like to be inclusive. With that, members were able to consider the perspectives of individuals with identities and experiences that differ from their own. These conversations revealed tensions and conflicting ideologies on what is inclusive or dismissive language given the context, offering the SLT an opportunity to build community and unify efforts to reform policies and align them with our values of diversity, equity, inclusion, and belonging. Rather than hold out for a perfect consensus, we were guided by our ACS partners to look for definitions that could pass a “scream test”; even if we had contradictory ideas for further optimization or emphasis, we could reach a stage where a definition did not make anyone want to scream.

The process of defining the terms proved to be just as important as the resulting definitions themselves. Crucially, the conversations that were part of our journey to articulating definitions were valuable, bringing light to ways that language is used and how decision-making could be more sensitive to the histories and preferences across DSPH. We also learned new technology to engage in conversations, in person, hybrid, and asynchronously. We in the Office of CCO also learned that it was better to come to SLT with an initial draft of language and ideas as opposed to blank canvas. We incorporated all of this learning in future activities such as developing mission and vision statements and developing CCO goals for the new DSPH strategic plan. Indeed, the enhanced ability to take the perspective of others in the school is likely an enduring benefit of the SLT convening’s, and that perspective taking stands to nurture the wisdom and creativity (6) to navigate a time of disruption.

## IDEA fellowship

The Impact and Development through Engagement and Advocacy (IDEA) Fellowship, previously known as the Inclusion, Diversity, Equity and Anti-Racism Fellowship, started when our first Associate Dean was appointed in 2020. The first two IDEA Fellows supported the work of the AAP and the AD, giving students the opportunity to gain knowledge and skills through experiential learning, supplemental to their studies at the university. Since 2020, the fellowship has evolved and expanded to a cohort of undergraduate and graduate students working with faculty and staff on selected health equity projects within DSPH. In the academic year 2024–2025, the Office of CCO selected its largest cohort to date with 13 students.

The goal of the IDEA Fellowship is to inspire collaboration between students, faculty and staff on public health practice, educational, or research projects at DSPH that promote and support work on equity, culture, and/or belonging. Students are paid 10 h per week for one academic year on a particular IDEA project. When applying to the IDEA Fellowship, students are asked to rank their top choice projects from a selection of projects submitted by faculty or staff mentors. If selected after the application and interview processes, students are matched to a specific project and their respective faculty or professional staff mentors. In addition to working with their mentors, IDEA Fellows meet as a cohort with activities facilitated by staff in the Office of CCO. These cohort meetings create a community to exchange ideas and support each other in their projects, professional development, and beyond [see Supplementary Data Sheet S1]. Furthermore, IDEA Fellows join the SLT, contributing student perspectives and input to guide decision-making around DEIB and CCO programming and goals at DSPH.

*“My journey with the Office of Diversity, Equity, Inclusion, and Belonging (DEIB) began as an Inclusion, Diversity, Equity, and Antiracism (IDEA) Fellow, analyzing the 2023 climate survey results and contributing to strategic initiatives for a more inclusive campus. That experience evolved into a research assistantship, where I helped revamp and redeploy the survey for its fourth iteration. My work with the office granted me a student membership on the DEIB shared leadership team. There, I received an unvarnished look at what it truly takes to drive systemic change—the messy, nonlinear work that rarely makes it into campus announcements”* Marie Owino, MPH Student and IDEA Fellow.

The Office of CCO has funded fellows through the TAE grant and an allocated DSPH budget. The fellows are chosen based on the number of proposals received, *availability* of funding, and project/student compatibility each year. Without the financial support of the TAE grant, we could not fund most of the students selected in previous years.

### Lessons learned

With guidance from the SLT and TAE, the IDEA Fellowship has expanded and improved its programming. Some members from the SLT serve as IDEA mentors, and all IDEA mentors are invited to be a part of the SLT for that year, providing direct feedback on the Fellowship’s effectiveness and challenges. Each year, IDEA fellows present their work to the SLT, sharing core insights and learnings from their experience. The SLT meetings also serve as a networking opportunity for students to engage with other faculty, staff and students on equity initiatives and explore further opportunities for cross-collaboration.

As the IDEA Fellowship expands, so does our growing cohort of students within the SLT. While this provides an opportunity to amplify the student’s voice, it also presents some challenges. As interest grows for the IDEA Fellowship, so do the application submissions and overall demand for funding and peoplepower to manage the process increases. Although the number of applications has grown each year, the number of selected applicants is limited by funding available each year; the number of projects/fellows has not been able to increase proportionally to the number of applications. Despite these challenges, the student’s voice is vital to the SLT, and the IDEA Fellowship program is an active part of achieving engagement and collaboration among students, staff and faculty; the IDEA Fellowship continues to be a priority within DSPH to ensure this legacy of shared leadership remains.

## Climate survey

The climate survey was initially created to be administered biennially and inform strategic direction within the DSPH community. In the climate survey, we ask participants about their perceptions of DSPH’s climate, how DSPH supports their success in their respective roles, how DSPH supports diversity, equity, inclusion, and belonging, and their personal experiences of faculty, staff and students within the environment at DSPH. Findings from the survey are used in strategy and decision-making conversations for DPSH. The Office of CCO continues administering a climate survey annually to monitor ongoing and emerging challenges and support the creation and expansion of an inclusive work environment that is aligned with DSPH mission and values.

The responses collected are confidential and de-identified in reports and presentations disseminated after data analysis. Aggregated results and summaries are shared with the SLT and with the school’s leaders across faculty, staff, and student governance structures. Data from this survey is used to develop strategies and actions to enhance the DSPH experience, policies, and environment.

The office partnered with TAE and Expanding the Bench through their Leaders in Equitable Evaluation and Diversity (LEEAD) scholars’ program to bring an evaluative and culturally responsive lens to redesign the climate survey. Through the LEAAD scholars’ program, evaluators from underrepresented backgrounds receive culturally responsive and equitable evaluation (CREE) training from seasoned evaluators. The LEEAD scholar who was matched with us joined us in redesigning the DPSH climate survey to align with the needs and objectives of the office, SLT, and DPSH at large.

*“As LEEAD scholar, I anchored the redesign of the climate survey and the definitions of DEIB collaboratively workshopped by the SLT and three CREE principles; 1) engagement of key stakeholders, 2) consideration of cultural and historical contexts, and 3) intentional data collection methods and practices. Before reworking the survey, it was essential to understand the historical context and cultural context of the school and the university, ensure that the survey included culturally responsive, and culturally relevant language. I leaned on the knowledge and expertise of the DEIB team to explore questions about the current university and school environment, past trends and results, and intended uses of the survey to understand the future goals and directions of the work. In these conversations, I worked to clarify the survey's purpose, what the DEIB office and SLT wanted to learn from the results, what aspects of diversity, equity, inclusion, and belonging to measure and uplift*.” Endaisia Love, LEEAD Scholar.

*“The SLT contributed to the recent 2025 DSPH climate survey along with the IDEA fellows, the LEAAD scholar, and me, as a student researcher of workplace culture. This collaboration enabled me to better understand the past surveys and results; it also gave me insight into the past climates of DSPH from staff and student perspectives. Input from multiple views led to the development of the most recent climate survey. This collaboration of insights from multiple positions within the school and from those with expertise on survey methodology and workplace culture shaped a comprehensive climate survey that really aimed to create a tool to understand the perceptions of climate at DSPH. The process of creating this survey helped me to better understand how the Office of DEIB not only valued input from all members of the community but also aims to incorporate this input into programming and other strategies.”* Tasha Joshua, DrPH Student.

*“The climate survey data revealed equally stark lessons about how data can tell a story—sometimes one that wasn’t always easy to hear. The survey responses revealed deep frustration: students of color reporting isolation, staff feeling siloed in their DEIB efforts, among other painful truths about inequities within our institution. But they also highlighted moments of hope: small wins, shifts in perspective, and the resilience of individuals who kept showing up. These efforts reinforced that the heart of this work is not policies or percentages, but people. The data was a call to action, a reminder that behind every statistic was someone’s lived experience.”* Marie Owino, MPH Student and IDEA Fellow.

### Lessons learned

The climate survey has evolved in themes, questions, and audience. It provides annual data on school morale, enabling leadership to identify strengths and challenges. The results underscore our shared responsibility to foster a safe, positive culture by strengthening engagement and collaboration across all levels of DSPH. We use these insights to uphold our commitment to an inclusive, engaging, and supportive environment for all. The climate survey has garnered lessons in how local and national climate impacts trust and participation. To acknowledge this, changes were made in this year’s survey with the addition of an emotion wheel that allowed participants to share how they were feeling during a time of uncertainty. This version of the survey offered participants the opportunity to provide more qualitative data along with the quantitative data. Moving forward, we plan to provide peer and leadership support to strengthen communication and encourage participation, to facilitate that the results more accurately reflect the full DSPH community and are more effectively disseminated to that community.

A lesson learned came from the dissemination of the climate survey results. On the surface, looking at some quantitative measures, it appeared that everything was fine and that we were doing a great job. However, when we dived deeper into stratified analyses by group (student, staff, and faculty) and individuals’ experiences through the analysis of open-ended responses, there was more than just a positive score on a Likert scale. We observed that, as a collective, we were doing well, but there were still areas where we needed to improve. What made this challenging to present was that some of the findings would contradict what some believed was possible within DSPH. This meant we not only had to present data elements but also be mindful of the cultural beliefs we would need to address. We were able to do this by establishing group agreements before presenting the data and by giving people space to explore their feelings about the data points.

## Sustainability efforts

Throughout our journey, we have navigated many internal and external changes that impact the policy and governance of our work. In response to the changing political environment, members of the office and the SLT have re-evaluated programming and efforts in compliance while exploring how to remain true to our mission and values. This process has been taxing, creating strain on systems and personnel. With so much noise and worry, focus is illusive, time seems fractured and scarce, and we fall short of consistently showing the traits we would like to model such as empathy (7) or a growth mindset (8). We second guess our use of words—diversity, equity, inclusion, and belonging—because of how they have been politicized, somehow yielding division when they have at their very core the opposite intention.

*“You may wonder if survival requires selling out, compromising the aspirations that brought you to where you are. I can say that from talking with other deans, both within public health and beyond, this is a cause of anguish among leadership, particularly in the past year. As we lead through a period of rapid change, windswept by forces we can shape only weakly and gradually, leaders in higher education walk a narrow path. A misstep in any direction could easily put at risk our own credibility, but even worse could jeopardize the mission-aligned work done by people for whom we have responsibility. I can easily lean too far into anticipatory compliance on one side, or to the side of inviting retaliation for speaking out…In the process of transforming academia for equity, even the term equity is up for debate. But then, language always changes. The source of hope that keeps me going is the determination I see in my colleagues at Drexel and across our TAE and ASPPH peers. I believe that our vision of a better future and our responsibilities to protect health will fuel us to stay in conversation. I believe that for each mistake that ruptures trust in ourselves or each other, there will emerge an opportunity to speak up, heal together by talking through what we have experienced, and then take the next step.”* Gina S. Lovasi, Dana and David Dornsife Dean, DSPH.

In maneuvering chaos, is there still a basis for optimism? Altering our language and office name warrant reflection and open discussion, as we work, and we protect intellectual exchange and education despite political polarization (9). In moving forward *with* this work, the office, SLT and leadership recognizes what we can do better, yet are limited in the funding we can invest and the time we can devote to aspirational change while also facing more immediate threats to survival. Beyond survival, there is a need to guard being true to who we are, so that we do not lose sight of either our origin or our forward-looking relevance. At Dornsife, we are a school founded on health as a human right, within a university founded on education in the practical arts and sciences offered regardless of background.

*“Perhaps we will find new solidarity in this time, where there previously was competition. Perhaps we will shed practices retained due to inertia that no longer do much to serve us and our students. Perhaps we can rethink how we leverage technology into our teaching and dissemination efforts so that we can personalize an engaging learning experience for our students, partners, and audiences. What gives me confidence that we can meet the challenge of this moment is the network that the RWJF funded Drexel Initiative for Transforming Academia for Equity (DITAE) has sustained at the Dornsife School of Public Health, and across the 7 grantee institutions. Seeing the persistence of our peer schools in very different state contexts has been a highlight of the annual TAE meetings I had an opportunity to join in 2024 and 2025*.” Gina S. Lovasi, DSPH Dean.

At annual TAE meetings, we benefit from hearing how other teams react to local constraints, which helped us recognize what we had taken for granted, and to probe what could change in the Commonwealth of Pennsylvania or nationally. We also heard clearly that the work of TAE was *hard*, scrappy, and yielded plenty of moments that were humbling, interspersed with the occasional joy of witnessing a breakthrough to reach a hard-won milestone.

In moving forward, an important part of our work is sustaining the SLT and institutionalizing our CCO efforts as a priority area with embedded goals in our strategic plan [see Supplementary Data Sheet S1]. Placing these within our school-wide strategic plan rather than a stand-alone action plan, like the AAP, would mean accountability for progress toward these goals during our annual state-of-the-school events. For this accountability, which has the potential to survive future leadership transitions if needed, having a few high-priority goals and metrics will serve us better than a list that is exhaustive. This is not only because of the distractions and survival threats we must navigate, but also because of the temptation to selectively report back on only those metrics which are easy to collect, or which paint a rosy picture, rather than all of those which are most important to fulfillment of our mission.

Our strategic planning includes goal setting *conversations* related to CCO with SLT and other members of DSPH. The work we had done to wrestle through definitions of diversity, equity, inclusion, and belonging will inform a refreshed way of articulating what we want our internal culture to be, and how we want that to be felt as a climate conducive to dignity, authenticity, creativity, and joy. Paired with the strategic plan, the Shared Leadership Team continues to grow and remain, creating a legacy of collective voice and advocacy.

*“Transitioning to a Shared Leadership Team (SLT) of over 55 individuals has brought its share of challenges—from logistical issues like scheduling to more complex questions such as how we define and reach consensus. Yet, I couldn’t be prouder of what we’ve built. We’ve cultivated a large, collegial community where openness, deep listening,* shared *vulnerability, and mutual celebration are part of our culture. This work is often slow-moving, frequently challenged, and, especially in today’s climate, sometimes discredited. Still, every time I engage with the SLT or the TAE community, I feel recharged, reenergized, and filled with joy. These moments strengthen my commitment to advancing this work and to fostering an environment at DSPH that embodies our shared vision of diversity, equity, inclusion, and belonging as we strive to realize health as a human right for all.”* Reneé H. Moore, Associate Dean, Office of CCO.

As individuals decide whether to sustain their engagement with the SLT, attention to our institutional incentives is warranted. Faculty annual reviews include opportunities to highlight aspects of service, teaching, and research with relevance to CCO, such as engagement as an IDEA Fellow mentor. Importantly, intrinsic motivation is also likely at play, with core beliefs driving our continued *engagement*. By showing up, we move closer to clarifying and reinforcing our commitments, both for ourselves and others, and affirm that we are acting in alignment with what we say we stand for. Sustained engagement can be jeopardized either by complacency and acceptance of the status quo, or by losing faith in our collective ability to make progress toward learning and improvement. To counter this, we hold and convey the view that improvements to our internal culture are needed, iterative, and achievable in ways that benefit our sense of community and improve access to opportunities regardless of role or background.

## Data Availability

The original contributions presented in the study are included in the article/Supplementary material, further inquiries can be directed to the corresponding author.
