# Supplementary material for: Shared leadership team: the creation, journey, lessons learned
Source: Front Public Health. 2026 Mar 17;14:1695469. doi: 10.3389/fpubh.2026.1695469 (PMC13035796; doi:10.3389/fpubh.2026.1695469)
Supplement: Supplementary file 1 [file Data_Sheet_1.docx]

**Appendix**

**Table 1.** DSPH Office of Culture, Community, and Opportunity’s Diversity Equity, Inclusion, and Belonging Definitions

| Diversity | A broad array of cultures, experiences, beliefs, ideas, ability, perspectives, at all levels and in all spaces in our school. This includes but is not limited to, race, ethnicity, gender, gender identity, language, age, sexual orientation, socioeconomic status, disability, religion, political beliefs, cultural background, and country of origin. |
| --- | --- |
| Equity | Fairness, access, opportunity, and advancement for all people while striving to identify and eliminate barriers that have prevented full participation. Equity recognizes that individuals and groups have different interests, needs, challenges, and starting points due to systemic inequalities. |
| Inclusion | Involves ensuring that people who have been excluded from important discussions historically have the opportunity to be fully involved in critical conversations. It is the practice of creating environments in which all individuals feel valued, respected, and actively engaged. We recognize that inclusion must be active and intentional, and it requires continuous collective reflections and learning throughout the system. |
| Belonging | Refers to the fundamental human need to feel accepted, valued, included, and connected within a group or community. It involves feeling supported an recognized as a full and authentic member of the Dornsife community who can contribute and receive freely and authentically, without needing to change or hide aspects of one’s identity. |

**Table 2**. DSPH Culture, Community, and Opportunity Strategic Planning Goals

| Goal 1: Amplify the Voices of All DSPH Members via Data Collection to identify strengths, address challenges, and provide recommendations that enhance culture and community |
| --- |
| Goal 2: Strengthen DSPH culture and wellbeing by expanding access to resources through strategic partnerships and training for students, staff, and faculty. |
| Goal 3: Foster a culture of shared decision making and strengthen community through shared leadership, of students, staff, and faculty. |

**CCO Goals finalized December 2025. Objectives and metrics to achieve these goals are being developed.*

**Table 3.** DSPH IDEA Fellowship Cohort Meeting Schedule Sample

| Cohort Meeting 1: Workplace Best Practices |
| --- |
| Cohort Meeting 2: Self Care in Practice |
| Cohort Meeting 3: Resume & Interview Skills |
| Cohort Meeting 4: Career Panel: Exploring Various Roles in Public Health |
| Cohort Meeting 5: Final Presentation of IDEA Fellowship Experience |

**Additional workshops are conducted per request such as Anti-Racist Practices in Research, Working with Community Partners, Qualitative Research Methods, Clinical Trials, Ethics & Data Management and more.*
